# Pulmonary Hemorrhage and Pneumothorax Risk During CT-Guided Lung Biopsy for Suspected Lung Cancer

**DOI:** 10.3390/cancers18050743

**Published:** 2026-02-26

**Authors:** Rosa Alba Pugliesi, Nour Maalouf, Giuseppe Gullo, Andreas H. Mahnken, Jonas Apitzsch

**Affiliations:** 1 Section of Radiology, Department of Biomedicine, Neuroscience and Advanced Diagnostics (BiND), University of Palermo, Via del Vespro 129, 90127 Palermo, Italy; 2 Department of Diagnostic and Interventional Radiology, Tϋbingen University Hospital, 72076 Tϋbingen, Germany; nour.maalouf@med.unituebingen.de; 3 Unit of Obstetrics and Gynecology, AOOR Villa Sofia Cervello, University of Palermo, 90100 Palermo, Italy; gullogiuseppe@libero.it; 4 Department of Diagnostic and Interventional Radiology, University Hospital of Marburg, 35043 Marburg, Germany; 5 Department of Radiology and Nuclear Medicine, Helios Hospital Pforzheim, 75175 Pforzheim, Germany; jonas.apitzsch@helios-gesundheit.de

**Keywords:** lung cancer, CT-guided lung biopsy, pneumothorax, pulmonary hemorrhage

## Abstract

Objectives: The aim of this study was to evaluate the association between pulmonary hemorrhage and PTX following CT-guided lung biopsy for suspected or confirmed lung malignancy and to assess whether lesion depth modifies this relationship. Methods: This retrospective single-center study included 118 consecutive patients undergoing CT-guided lung biopsy for oncologic indications between 2020 and 2025 (66 men, 52 women; median age 69 years). Immediate post-biopsy CT was assessed for PTX and focal pulmonary hemorrhage. Multivariable logistic regression identified predictors of PTX, including pulmonary hemorrhage, lesion size and depth, chronic obstructive pulmonary disease, patient positioning, age, and sex. An interaction analysis evaluated effect modification by lesion depth. Results: PTX occurred in 22.0% of biopsies, and pulmonary hemorrhage in 29.7%. PTX was significantly less frequent in patients with pulmonary hemorrhage (*p* = 0.021). Lesion depth showed a borderline association with PTX risk (OR 1.02 per mm; *p* = 0.060). Pulmonary hemorrhage demonstrated a nonsignificant protective trend (OR 0.33; *p* = 0.135). The hemorrhage–depth interaction approached significance (*p* = 0.065), suggesting attenuation of depth-related PTX risk. Model discrimination was moderate (AUC = 0.709). Conclusions: In patients undergoing CT-guided biopsy for lung cancer evaluation, pulmonary hemorrhage may mitigate PTX risk, particularly for deeper lesions.

## Simple Summary

CT-guided lung biopsy is widely used to diagnose suspected lung cancer and to guide treatment decisions such as surgery, radiotherapy, and immunotherapy. Pneumothorax, a collapse of the lung caused by air leakage, is the most common complication of this procedure. Pulmonary hemorrhage, which appears as localized bleeding around the biopsy site, is usually considered an adverse event. In this retrospective study of 118 patients undergoing CT-guided lung biopsy for suspected or confirmed lung cancer, we found that pneumothorax occurred less frequently in patients who developed pulmonary hemorrhage. Our results suggest that limited hemorrhage may partially seal the biopsy tract and reduce air leakage, especially in deeper lung lesions. Understanding this interaction may help improve biopsy safety, reduce procedure-related delays, and support timely initiation of cancer treatments.

## 1. Introduction

Lung cancer is the predominant cause of cancer-related mortality worldwide, leading to a rising demand for minimally invasive diagnostic techniques, with more than two million new cases diagnosed annually [1]. The widespread implementation of molecular profiling and precision oncology has markedly increased the incidence of CT-guided lung biopsies performed for initial diagnosis and treatment stratification [2]. Consequently, improving the safety profile of CT-guided transthoracic needle biopsy (TTNB) has become a clinically important objective [3].

TTNB is a crucial minimally invasive technique for detecting pulmonary nodules, masses, and ground-glass opacities in individuals with suspected lung cancer [4]. The precise histological and molecular characterization obtained by TTNB is essential for personalized cancer treatment, guiding the selection and sequence of radiation, immunotherapy, targeted therapy, and surgical interventions [5]. In precision oncology, procedural challenges may obstruct therapy or limit appropriate treatment options, making the safety and reliability of diagnostic techniques crucial [6]. Although pulmonary hemorrhage typically manifests as perilesional or intrapulmonary bleeding and often resolves spontaneously, pneumothorax (PTX) may necessitate chest tube placement and prolonged hospitalization, potentially complicating cancer staging, radiation planning, or surgical interventions [7,8].

Given that numerous patients qualify for multimodal treatment strategies encompassing surgery, radiation, and immunotherapy, mitigating biopsy-related complications is particularly crucial within the framework of multidisciplinary lung cancer management [9]. Delays or cancellations caused by PTX may negatively impact treatment schedules and overall cancer results. Traditionally, PTX and pulmonary hemorrhage have been regarded as independent complications; however, emerging evidence suggests a possible inverse relationship between them [10,11,12,13]. Immediate post-biopsy pulmonary bleeding has been linked to decreased PTX rates [14], suggesting that it may seal the biopsy tract and reduce alveolar air leakage [4,11,12].

Deeper, peripheral lesions, commonly targeted for stereotactic radiation or minimally invasive surgical excision, have a higher intrinsic risk of PTX, making this protective relationship of relevance in oncologic imaging. Determining if pulmonary bleeding alters this risk could guide biopsy planning procedures that facilitate subsequent cancer therapy.

Unlike our prior studies [15,16,17], which concentrated on technical standardization, needle angulation within an 80–100° safe zone and organized post-biopsy monitoring protocols to mitigate PTX incidence, the current study examines whether pulmonary hemorrhage may biologically influence PTX risk.

While demographic factors, COPD status, lesion size, patient positioning, and procedural standardization have already been systematically evaluated in our prior publications [15,16,17], the current analysis specifically explores the interaction between pulmonary hemorrhage and lesion depth in an oncologic cohort. Rather than re-examining established predictors, this study was designed to investigate whether tract-related hemorrhage exerts a potential tamponade-like effect that attenuates depth-associated PTX risk.

A methodologically oriented study that emphasized predictive modeling and risk stratification of biopsy complications has previously analyzed a partially overlapping cohort from our institution [18]. In contrast, the current study examines the relationship between pulmonary hemorrhage and PTX, with a focus on a possible depth-dependent protective interaction, in order to answer a clinical and mechanistic question. Rather than enhancing predictive algorithms, the objective of the present study is to offer clinically interpretable insights into complication mechanisms that are pertinent to cancer treatment workflows and procedural decision-making.

Bronchoscopic procedures, although associated with lower PTX risk, demonstrate lower diagnostic accuracy than TTNB for peripheral pulmonary lesions, including early-stage lung cancers or oligometastatic disease [19]. TTNB is crucial in lung cancer management, emphasizing the necessity for improved risk classification and complication mitigation [20,21].

This study explores the correlation between pulmonary hemorrhage and PTX in individuals undergoing oncologic CT-guided lung biopsy. We hypothesized that pulmonary hemorrhage reduces the depth-related risk of PTX, thereby enabling safer diagnostic access to lesions pertinent for radiotherapy targeting, immunotherapy decision-making, and surgical planning. Using multivariable logistic regression, we sought to characterize this relationship and contribute clinically meaningful data, including borderline and negative findings, to the optimization of diagnostic pathways in lung cancer therapy. Although COPD and other established predictors were not the primary focus of this investigation, they were incorporated into the regression model as adjustment variables. Additionally, exploratory subgroup analyses were performed to assess whether the hemorrhage–PTX relationship differed within clinically relevant subpopulations such as patients with COPD.

## 2. Materials and Methods

A retrospective single-center cohort study was performed in 118 consecutive patients (66 men, 52 women; median age: 69 years, range: 49–90 years) who had undergone CT-guided transthoracic lung biopsy between 9 January 2020 and 4 April 2025. All biopsies were performed by a single board-certified interventional radiologist with 18 years’ experience, minimizing inter-operator variation. The study was approved by the Institutional Ethics Committee of the Landesärztekammer Baden-Württemberg, Germany (approval number F-2021-038), and all procedures were conducted in accordance with the Declaration of Helsinki. All participants were informed about the intervention more than 24 h in advance and provided written informed consent.

Patients were sampled from an institutional radiology database by procedural code for CT-guided biopsies. All patients with adequate follow-up imaging and in the absence of a pre-existing chest tube were considered for inclusion. COPD was defined based on clinical diagnosis corroborated by imaging evidence of emphysema. Spirometric data were available for 62% of patients; where absent, imaging features (centrilobular or paraseptal emphysema) were used as surrogate criteria.

Data collected included demographics (age, sex), lesion features (size in mm, distance from pleural surface in mm), and presence of existing COPD. Histopathological diagnoses were derived from pathology reports. Only pathology records relating to CT-guided biopsy were used.

Only intrapulmonary lesions were included; mediastinal or pleural-based targets were excluded. All cases were evaluated pre-procedurally by a thoracic radiologist and classified as unsuitable for bronchoscopy due to peripheral location, absence of bronchial access, or lesion size. Biopsies were performed for suspected malignancy, metastatic lesions, or atypical infectious/inflammatory disease.

Standardized definitions were applied: PTX was defined as any pleural air collection visible on immediate or delayed post-biopsy CT; pulmonary hemorrhage as high-attenuation perilesional or intrapulmonary opacities on immediate post-biopsy CT; chest drainage as chest tube placement performed immediately after the procedure or during hospitalization; and needle path length as the manually measured distance from the pleural puncture site to the lesion center on CT. The primary outcome was PTX (yes/no), the key independent variable was pulmonary hemorrhage (yes/no), and secondary outcomes included the need for chest drainage as well as the interaction between hemorrhage and lesion depth.

Given that COPD status, patient positioning, demographic variables, and technical standardization effects have been comprehensively analyzed in our previous institutional studies, the present investigation did not aim to re-establish these predictors. Instead, these variables were included in the multivariable model as adjustment covariates to isolate the independent association between pulmonary hemorrhage and PTX.

No formal sample size calculation was performed prior to data collection due to the retrospective design. With 26 PTX events among 118 patients, the number of outcome events relative to included covariates may limit statistical power, particularly for interaction testing. This is acknowledged as a methodological limitation. In addition to multivariable modeling, subgroup analyses were performed in patients with COPD to evaluate whether the association between pulmonary hemorrhage and PTX differed within this high-risk population. These analyses were considered exploratory due to limited sample size.

### 2.1. Biopsy Needle and Trocar

An 18G semi-automatic Tru-Cut biopsy needle with a notched inner stylet and sliding outer sheath was used through a 17G coaxial trocar (Möller Medical GmbH, Fulda, Germany). The coaxial system permitted multiple tissue samples through a single pleural puncture, minimizing trauma and improving diagnostic yield.

### 2.2. Biopsy Protocol

All procedures followed a standardized protocol including sterile preparation, local anesthesia with 1% mepivacaine, and a single pleural puncture. Needle trajectory was planned using pre-biopsy imaging and executed with the patient in prone or supine position depending on lesion location. Sequential CT guidance (64-slice Siemens Somatom Edge, 2 mm slice thickness, end-expiratory breath-hold, no contrast) was used to confirm needle placement. A single pleural pass was targeted in all cases, with the needle placed as perpendicular to the pleura as feasible. Biopsy duration was kept under six minutes. No prophylactic blood patches were used. Tissue samples were fixed in formaldehyde and sent for histopathology.

All patients underwent immediate post-biopsy low-dose CT, acquiring just a short z-axis volume of the area biopsied, to detect complications. Follow-up imaging within 7 days was performed if patients developed new symptoms or signs suggestive of delayed complications.

### 2.3. Statistical Analysis

Continuous variables were presented as mean ± standard deviation (SD) or median (range), while categorical variables were presented as counts and percentages. Association between hemorrhage and PTX was tested by Chi-square or Fisher’s exact tests for categorical variables and *t*-tests or Mann–Whitney U tests for continuous variables, based on distribution.

A multivariable logistic regression model was constructed with PTX as the outcome variable. Independent predictors included pulmonary hemorrhage, lesion size, lesion depth (needle path), COPD status, age, gender, and biopsy position. An interaction term between hemorrhage and lesion depth, representing whether the effect of depth on PTX risk differs depending on the presence or absence of hemorrhage, was included to test for effect modification. All predictors were entered simultaneously in the multivariable model.

Model performance was evaluated using the area under the ROC curve (AUC), and multicollinearity was assessed via variance inflation factors (VIF). Sensitivity analysis was conducted by excluding statistical outliers (i.e., very deep or very large lesions).

No formal sample size calculation was performed prior to data collection; however, with 26 PTX events in a sample of 118, the study had sufficient power for approximately five covariates; however, seven variables were explored based on clinical relevance, acknowledging possible model overfitting as a limitation.

A two-sided *p*-value < 0.05 was considered statistically significant. Analyses were performed using R software (version 4.5.0).

## 3. Results

A total of 118 patients were included in this study. CT-guided biopsies were performed in the supine position in 65 patients (55%) and in the prone position in 53 patients (45%). Thirty-nine patients (33%) had a prior diagnosis of COPD. The mean lesion size was 38.6 ± 26.6 mm (range: 2.6–115 mm), and the mean lesion depth (needle path length) was 24.1 ± 16.9 mm.

A PTX occurred in 26 patients (22%), of whom 7 (27%) required immediate chest tube placement. An additional 8 patients (7%) required chest drainage during the clinical course. Pulmonary hemorrhage occurred in 35 patients (30%).

Among patients with PTX, 73% did not experience hemorrhage, while 27% experienced both complications (Table 1). This descriptive distribution suggests, but does not statistically demonstrate, an inverse trend between hemorrhage and PTX.

Excluding statistical outliers (lesion depth > 70 mm or size > 90 mm) yielded similar results: hemorrhage OR 0.48 (*p* = 0.27); COPD remained statistically significant (OR 3.12, *p* = 0.041); AUC = 0.71.

### 3.1. Lesion Size Analysis

Lesion sizes in the population under study ranged from 2.6 mm to 115 mm. The mean lesion size among patients with PTX was 33.3 mm, while among patients without PTX had a mean lesion size of 40.1 mm. This difference was not statistically significant (*p* = 0.221, Mann–Whitney U test).

### 3.2. Lesion Depth and Hemorrhage Interaction

Needle path length ranged from 0 to 80 mm. Logistic regression showed that greater needle depth was significantly associated with increased PTX risk (β = 0.033, *p* = 0.012). Hemorrhage demonstrated a non-significant association with PTX (β = 1.103, *p* = 0.191). The interaction term between hemorrhage and needle depth approached significance (β = −0.047, *p* = 0.065), suggesting that hemorrhage may moderate the effect of needle depth on PTX risk.

**Figure 1:**
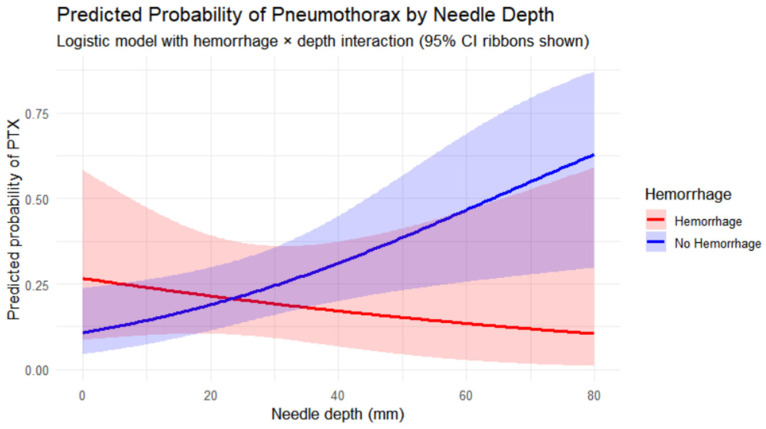
Interaction between needle depth and pulmonary hemorrhage on PTX risk. Logistic regression demonstrated a significant effect of needle depth (*p* = 0.012), a non-significant main effect of hemorrhage (*p* = 0.191), and a near-significant interaction term (*p* = 0.065). Shaded areas represent 95% confidence intervals.

Model-predicted risks of PTX increased rapidly with deeper needle depth in the absence of hemorrhage. In contrast, the presence of hemorrhage appeared to blunt this depth-associated risk increase, supporting a potential protective interaction. These are shown graphically in Figure 1, where diverging risk curves with depth can be observed over the range of depths. The overall model fit was acceptable (residual deviance = 117.10 on 114 degrees of freedom; AIC = 125.1).

Predicted PTX risks were estimated for lesions at 10-, 20-, and 40 mm depths: without hemorrhage = 8%, 17%, 32%; with hemorrhage = 7%, 10%, 15%, respectively, indicating attenuation of depth-related risk.

### 3.3. Complication Interaction

**Figure 2:**
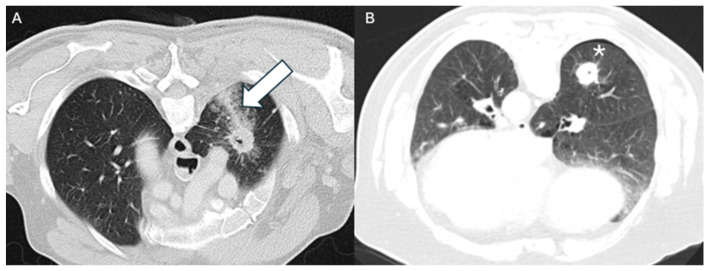
Representative axial CT images demonstrating the inverse relationship between pulmonary hemorrhage and PTX. (**A**) Axial CT image (lung window, 2 mm slice thickness, 1 mm increment) shows the intrapulmonary lesion with perilesional hemorrhage along the needle trajectory (arrow). No PTX is visible. (**B**) Corresponding CT image demonstrates the intrapulmonary lesion without adjacent hemorrhage. A narrow PTX is present along the needle path (asterisk).

**Figure 3:**
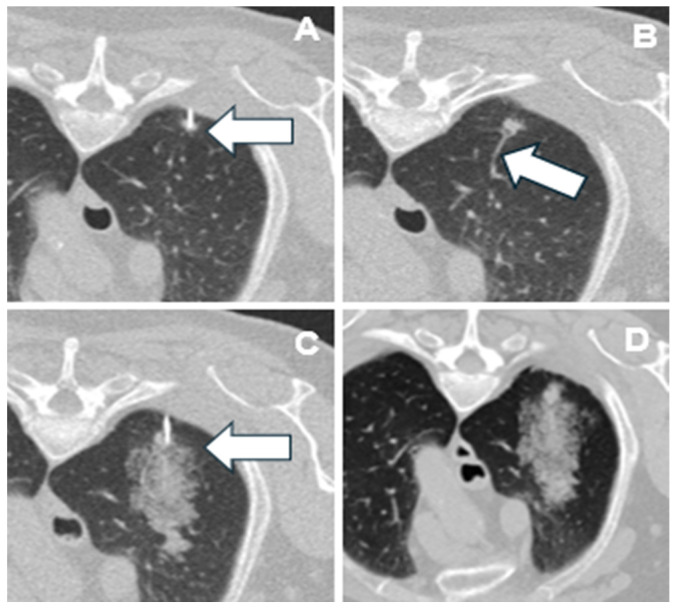
Representative sequential CT images illustrating biopsy-related vessel injury with subsequent tract hemorrhage. (**A**) Initial image showing the biopsy needle accurately positioned within a very small pulmonary nodule (arrow). (**B**) Image depicting a feeding vessel (arrow) supplying the nodule, which appears disrupted as a result of the biopsy. (**C**) Hemorrhage is visible along the course of the injured vessel (arrow), while the needle remains confined within the nodule, indicating that the hemorrhage was not due to parenchymal trauma from the needle itself. (**D**) Immediate post-procedural control CT obtained at 14:57, three minutes after the procedure concluded at 14:54, confirming the presence of hemorrhage as a biopsy-related complication.

Hemorrhage was significantly less frequent in patients who developed PTX in unadjusted analysis (*p* = 0.021), whereas in the multivariable model this association did not reach statistical significance (OR 0.33, *p* = 0.135). Chest tube placement occurred in 3/35 (9%) patients with hemorrhage versus 12/83 (14%) without hemorrhage; this difference was not statistically significant (*p* = 0.48, Fisher’s exact test). Fewer patients with hemorrhage required chest tubes, although the difference was not statistically significant. These findings suggest a potential protective effect of hemorrhage (Figure 2), possibly via local tamponade or sealing mechanisms [22], as illustrated in Figure 3, which provides a case example showing the development of hemorrhage following vessel injury during needle placement, supporting a biologically plausible pathway for this protective effect.

The image sequence demonstrates that hemorrhage originated from a disrupted feeding vessel, not from parenchymal trauma, while the needle remained in place. Hemorrhage from a small vessel may exert a local tamponade effect, promoting clot formation along the needle tract, thereby reducing air leakage and the likelihood of PTX formation. Similar mechanisms have been postulated in prior interventional studies, where intrapulmonary bleeding or tract embolization was shown to decrease post-biopsy PTX by sealing the puncture channel [23,24,25]. Conversely, diffuse parenchymal trauma produces air leakage without effective sealing, a phenomenon also described in experimental models of alveolar injury and barotrauma [26,27].

### 3.4. Logistic Regression Predictors and Model Performance in PTX Risk Prediction

A multivariable logistic regression model was constructed to evaluate predictors of PTX, including pulmonary hemorrhage, lesion size, lesion depth, COPD, age, and gender. VIF values were all below 2, indicating no evidence of multicollinearity among the included variables.

Two variables emerged as statistically significant predictors of PTX risk:

- Lesion size: OR 0.97 per mm increase (95% CI: 0.95–0.99, *p* = 0.020).
- COPD: OR 3.17 (95% CI: 1.08–9.48, *p* = 0.035).

Lesion depth showed borderline association (*p* = 0.060). Hemorrhage and the interaction term did not reach statistical significance.

**Figure 4:**
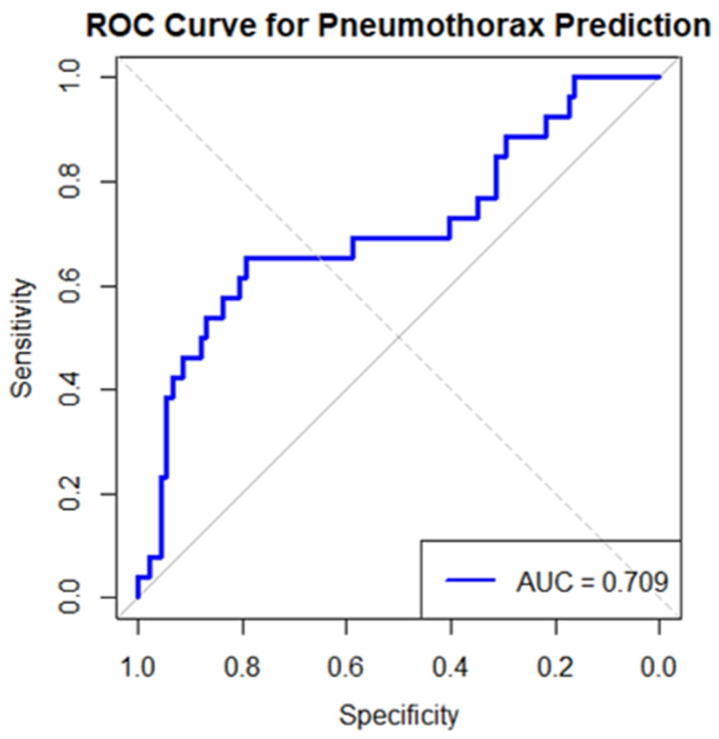
Multivariable model for PTX prediction after CT-guided lung biopsy. Receiver operating characteristic (ROC) curve from the logistic regression model including hemorrhage, lesion size, lesion depth, COPD, age, and gender (AUC = 0.709; moderate discrimination). The solid line represents model performance; the dotted diagonal indicates no-discrimination (chance) reference.

Model performance is illustrated in Figure 4, which displays the ROC curve for PTX prediction. The model achieved an AUC of 0.709 (95% CI: 0.61–0.81), indicating moderate discriminatory ability.

Lesion size and COPD remained the strongest predictors within the multivariable model, whereas hemorrhage, age, and gender were not statistically significant in this cohort. Full regression results, including odds ratios and confidence intervals, are presented in Table 2.

### 3.5. COPD and Risk Profile

**Figure 5:**
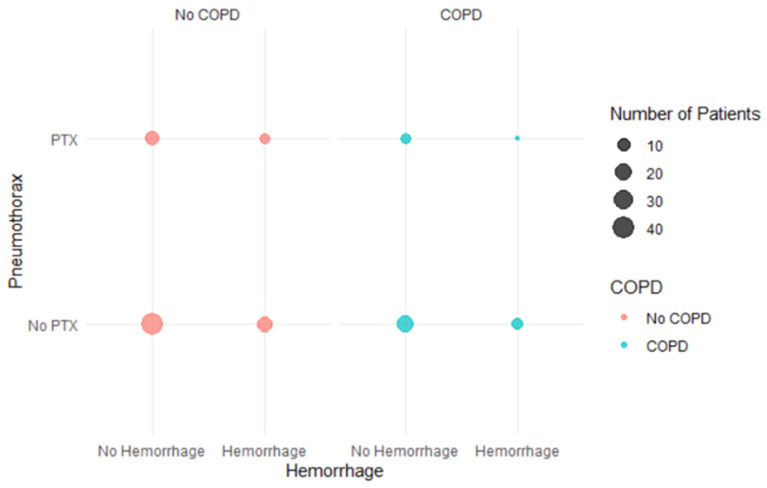
Balloon plot showing the relationship between pneumothorax (PTX) and hemorrhage, stratified by COPD status. Each bubble represents a group defined by presence or absence of hemorrhage and PTX. Bubble size corresponds to the number of patients in each group, while color indicates COPD status: red for patients without COPD and cyan for those with COPD. The distribution shows no significant association between hemorrhage and PTX among COPD patients (Fisher’s exact test, *p* = 0.653).

As shown in the multivariable model (Table 2), COPD independently predicted PTX risk (OR 3.17, *p* = 0.035). Within the COPD subgroup (*n* = 39), PTX occurred in 7/39 (18%). PTX occurred in 6/29 (21%) without hemorrhage versus 1/10 (10%) with hemorrhage (Fisher’s exact *p* = 0.653). However, by Fisher’s exact test, there was no statistically significant association between hemorrhage and PTX in COPD patients (*p* = 0.653), with an estimated odds ratio of 0.43 (95% CI: 0.01–4.45). This suggests a trend towards lower risk of PTX in hemorrhage cases, although with limited interpretation with small sample size and wide confidence intervals. Pearson’s chi-square tests, with and without continuity correction, also confirmed the lack of statistical significance (*p* = 0.45 and *p* = 0.78, respectively). Figure 5 visualizes the subgroup counts for COPD patients, highlighting these distributions without displaying regression coefficients.

### 3.6. Histological Outcomes

**Figure 6:**
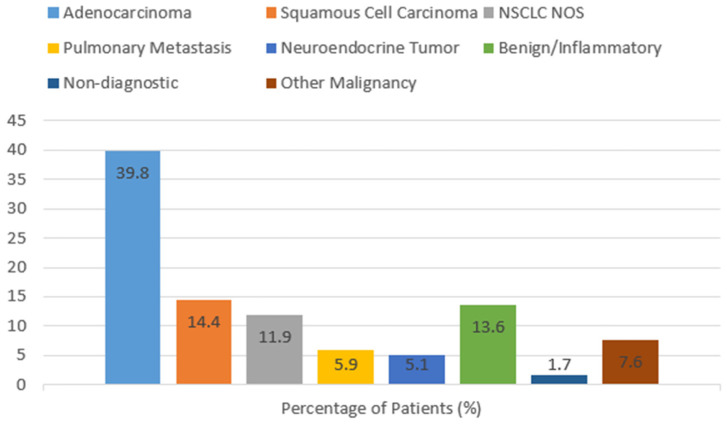
Distribution of histological diagnoses among biopsied pulmonary lesions. Bar chart showing the distribution of histological diagnoses with corresponding percentages. NSCLC NOS indicates non-small-cell lung carcinoma not otherwise specified. The Benign/Inflammatory group comprises non-neoplastic findings, including organized pneumonia, chronic inflammatory changes, and pulmonary fibrosis.

Histopathological evaluation was completed successfully in 116 (98.3%) of 118 patients but 2 (1.7%) were described as non-diagnostic with no satisfactory material or no tumor cells available. Of the cases diagnosed, 77.6% were malignancies. The most prevalent malignant type was adenocarcinoma, which was found in 39.8% of patients. This was followed by squamous cell carcinoma (14.4%), NSCLC NOS at 11.9%, and pulmonary metastases at 5.9%. Neuroendocrine tumors, including carcinoids and atypical ones, accounted for 5.1%. Other malignancies, such as sarcoma and fibrous tumors, comprised 7.6%. Benign and inflammatory lesions were 13.6% of the cases, i.e., organized pneumonia, chronic inflammation, fibrosis, granulomatous diseases, and hamartomas. Figure 6 illustrates the frequencies of histological findings.

Because several histological subgroups contained limited numbers of cases, diagnosis-specific risk analyses for PTX or pulmonary haemorrhage were not performed. Therefore, histological outcomes are presented descriptively and were not included as predictors in multivariable modeling.

## 4. Discussion

In an oncological population, this retrospective single-center cohort study examined the correlation between PTX and pulmonary hemorrhage subsequent to CT-guided transthoracic lung biopsy. Our data suggest that pulmonary hemorrhage may be associated with a reduced incidence of PTX, particularly in cases of increased lesion depth. This implies a tamponade-like mechanism in which PTX is inhibited by localized hemorrhage from the biopsy tract, which reduces the influx of alveolar air into the pleural cavity [17,28,29]. This method is advantageous in the management of lung cancer, as it frequently requires tissue confirmation for radiation therapy, immunotherapy, and surgical intervention, particularly for the treatment of larger and peripheral lesions.

The 22.0% PTX rate in our sample corresponds with the rates reported in extensive meta-analyses utilizing CT-guided transthoracic needle biopsy, which range from 15% to 30%, depending on patient selection, lesion characteristics, and technical methodologies [30]. Chest tube implantation rates in documented research typically range from 5% to 15% [31], consistent with our cohort’s findings, where 5.9% required immediate drainage, and an additional 6.8% requiring further chest tube insertion. This consistency reinforces the external validity of our findings and indicates that the identified interaction patterns between bleeding and lesion depth are improbable to represent unusual complication rates.

From a clinical management standpoint, these complication rates highlight the necessity of doing a comprehensive procedural risk assessment, since hospitalization or intervention due to PTX may postpone cancer staging, radiation planning, or the commencement of systemic therapy.

Numerous constraints merit attention. The retrospective design limits causal inference and creates potential selection and information biases. Despite all procedures being conducted by a solitary, proficient interventional radiologist, thereby minimizing technical variability, the single-center context may restrict generalizability. The small sample size, especially the restricted number of PTX occurrences, hampered statistical power for subgroup analyses, including those involving patients with COPD. Consequently, these subgroup findings should be regarded as exploratory and necessitate validation in larger prospective multicenter datasets.

Unmeasured confounders, such as comprehensive smoking history, quantitative emphysema burden, and specific procedural factors, may have affected the probability of complications [32]. Future research utilizing standardized clinical datasets, quantitative imaging biomarkers, or radiomic factors may enhance personalized risk modeling for patients undergoing lung biopsy. Moreover, despite the documentation of histological diagnoses, the subgroup numbers were inadequate to provide a reliable comparison of PTX or bleeding risk across distinct tumor types. Consequently, diagnosis-specific odds ratio studies were not conducted and should be examined in larger multicenter cohorts.

The study’s strengths comprise a consistent procedural technique executed by a solitary experienced interventional radiologist, standardized biopsy protocols involving a single pleural pass, and a systematic immediate post-biopsy CT assessment, which collectively minimized procedural variability and facilitated a concentrated evaluation of hemorrhage-pneumothorax interactions.

Notwithstanding these constraints, the multivariable model exhibited considerable discriminative efficacy (AUC = 0.709), aligning with previous studies that recognize lesion depth, lesion size, and COPD as significant predictors of PTX risk [33,34]. The interaction between pulmonary hemorrhage and lesion depth did not achieve statistical significance but indicated a potential depth-dependent protective effect that warrants further exploration. This approach is especially pertinent in cancer, where deeper lesions are frequently targeted to facilitate definitive treatment techniques such as stereotactic radiation therapy, minimally invasive resection, or systemic therapy [35].

Reconceptualizing pulmonary bleeding as a potential modulator instead of merely an undesirable event could influence post-biopsy care. Identifying restricted tract bleeding on initial imaging may guide customized monitoring techniques and discharge planning, thereby decreasing unnecessary hospitalizations and eliminating delays in oncologic therapy [36]. Moreover, these findings offer conceptual validation for novel preventive strategies, such as tract embolization procedures, which similarly seek to occlude the biopsy channel and diminish the incidence of PTX [18,37,38]. Our findings collectively advance procedural risk models aimed at improving the safety of diagnostic techniques critical to modern lung cancer treatment.

## 5. Conclusions

In patients undergoing CT-guided lung biopsy for lung cancer evaluation, pulmonary hemorrhage may decrease the likelihood of pneumothorax, particularly in cases involving lesions located deeper within the lung tissue. By potentially reducing PTX-related morbidity and the need for invasive interventions, this interaction may help preserve procedural safety and minimize delays in diagnosis-dependent treatment processes. Ensuring the safety of biopsies is essential for the timely initiation of radiotherapy, immunotherapy, and surgical interventions, all of which depend on accurate and efficient tissue diagnosis. Although constrained by its retrospective single-center design and moderate sample size, this study offers clinically significant insights into the interplay of biopsy-related complications within an oncologic setting. Prospective multicenter studies are necessary to confirm these findings and to further enhance risk stratification models that facilitate efficient, therapy-focused diagnostic workflows in lung cancer management.

## Figures and Tables

**Table 1:** Summary of Procedure Characteristics and Complications. Baseline demographic characteristics, lesion parameters (mean ± standard deviation and range), and procedure-related complication rates in 118 patients undergoing CT-guided transthoracic lung biopsy.

Variable	Value
Total patients	118
Male/Female	66 (56%)/52 (44%)
Median age	69 years (range: 49–90)
COPD	39 (33%)
Mean lesion size	38.6 ± 26.6 mm (range: 2.6–115 mm)
Mean lesion depth	24.1 ± 16.9 mm (range: 0–80 mm)
Pneumothorax	26 (22%)
Immediate chest tube placement	7 (6%)
Chest tube during clinical course	8 (7%)
Pulmonary hemorrhage	35 (30%)

**Table 2:** Multivariable logistic regression analysis of factors associated with PTX after CT-guided lung biopsy. Lesion size and COPD status were significant predictors. The interaction between pulmonary hemorrhage and lesion depth showed a trend toward significance, suggesting a potential moderating effect. Reference categories: no hemorrhage, no COPD, female gender, supine position. OR = Odds Ratio; CI = Confidence Interval. * Statistically significant (*p* < 0.05); † Trend toward significance (*p* < 0.1).

Predictor	OR	95% CI	*p*-Value
Pulmonary Hemorrhage (yes vs. no)	0.33	0.08–1.44	0.135
Lesion Size (per mm increase)	0.97	0.95–0.99	0.020 *
Lesion Depth (per mm increase)	1.02	1.00–1.05	0.060
COPD (yes vs. no)	3.17	1.08–9.48	0.035 *
Age (per year increase)	1.01	0.96–1.07	0.670
Gender (male vs. female)	1.09	0.42–2.84	0.860
Patient Position (prone vs. supine)	1.28	0.50–3.28	0.610
Hemorrhage × Lesion Depth (interaction)	0.95	0.90–1.00	0.065 †

## Data Availability

The data presented in this study are openly available in FigShare at Pugliesi, Rosa Alba; Christoph Apitzsch, Jonas (2025). Dataset study—Impact of Technical Standardization on Pneumothorax and Chest Tube Insertion Rates: A Retrospective Learning Curve Analysis of CT-Guided Lung Biopsies. figshare. Dataset. https://doi.org/10.6084/m9.figshare.28925330 (accessed on 23 February 2026).

## Abbreviations

The following abbreviations are used in this manuscript:

CT	Computed Tomography
TTNB	Transthoracic Needle Biopsy
PTX	Pneumothorax
COPD	Chronic Obstructive Pulmonary Disease
AUC	Area Under the Curve
ROC	Receiver Operating Characteristic
SD	Standard Deviation
OR	Odds Ratio
CI	Confidence Interval
VIF	Variance Inflation Factor
EPV	Events Per Variable

## References

[1] Chen H.H., Wu Y.J., Wu F.Z. (2025). Precision Medicine in Lung Cancer Screening: A Paradigm Shift in Early Detection-Precision Screening for Lung Cancer. Diagnostics.

[2] Tang F.H., Wong H.Y.T., Tsang P.S.W., Yau M., Tam S.Y., Law L., Yau K., Wong J., Farah F.H.M., Wong J. (2025). Recent advancements in lung cancer research: A narrative review. Transl. Lung Cancer Res..

[3] Poulou L.S., Tsagouli P., Ziakas P.D., Politi D., Trigidou R., Thanos L. (2013). Computed tomography-guided needle aspiration and biopsy of pulmonary lesions: A single-center experience in 1000 patients. Acta Radiol..

[4] Sridhar S., Ahn H.G., Ebrahimzadeh S., Tang F., Elicker B. (2025). Evidence-based approach to transthoracic needle biopsy: Procedural techniques, risks, and controversies. RadioGraphics.

[5] Riedl J.M., Moik F., Esterl T., Kostmann S.M., Gerger A., Jost P.J. (2024). Molecular diagnostics tailoring personalized cancer therapy—An oncologist’s view. Virchows Arch..

[6] Constantinescu A., Stoicescu E.R., Iacob R., Chira C.A., Cocolea D.M., Nicola A.C., Mladin R., Oancea C., Manolescu D. (2024). CT-Guided Transthoracic Core-Needle Biopsy of Pulmonary Nodules: Current Practices, Efficacy, and Safety Considerations. J. Clin. Med..

[7] Wattanasatesiri T., Puntu W., Vithitsuvanakul N. (2018). Influencing factors of pneumothorax and parenchymal hemorrhage after CT-guided transthoracic needle biopsy: Single-institution experience. Pol. J. Radiol..

[8] Huang Z.G., Sun H.L., Wang C.L., Gao B.X., Chen H., Yang M.X., Chen X.L. (2021). CT-guided transthoracic needle biopsy of pulmonary lesions: Comparison between the cutting needle and aspiration needle. Br. J. Radiol..

[9] Brönnimann M.P., Manser L., Christe A., Heverhagen J.T., Gebauer B., Auer T.A., Schnapauff D., Collettini F., Schroeder C., Dorn P. (2025). Ground-glass opacities in the access route and biopsy in highly perfused dependent areas of the lungs as risk factors for pulmonary hemorrhage. Tomography.

[10] Wang Y., Zhang Y., Ren N., Li F., Lu L., Zhao X., Zhou Z., Gao M., Wang M. (2024). Repeat biopsy versus initial biopsy for non-small cell lung cancer: Complication risk factors and outcomes. Front. Oncol..

[11] Zhu J., Qu Y., Wang X., Jiang C., Mo J., Xi J., Wen Z. (2020). Risk factors associated with pulmonary hemorrhage and hemoptysis following percutaneous CT-guided transthoracic lung core needle biopsy: A retrospective study of 1090 cases. Quant. Imaging Med. Surg..

[12] Zhou S.Q., Luo F., Li K., Ran X., Lv F.R. (2023). Association between needle track bleeding and immediate pneumothorax in CT-guided lung biopsies. Sci. Rep..

[13] Chan M.V., Afraz Z., Huo Y.R., Kandel S., Rogalla P. (2023). Manual aspiration of a pneumothorax after CT-guided lung biopsy: Outcomes and risk factors. Br. J. Radiol..

[14] Bingham B.A., Huang S.Y., Chien P.L., Ensor J.E., Gupta S. (2020). Pulmonary Hemorrhage Following Percutaneous Computed Tomography-Guided Lung Biopsy: Retrospective Review of Risk Factors, Including Aspirin Usage. Curr. Probl. Diagn. Radiol..

[15] Pugliesi R.A., Nasser Y., Benchekroun A., BenAyed R., Mahnken A.H., Maalouf N., Apitzsch J. (2025). Impact of technical standardization on pneumothorax and chest tube insertion rates. J. Clin. Med..

[16] Maalouf N., Abou Mrad M., Benayed R., Pugliesi R.A., Apitzsch J. (2026). Time and angle interplay with pneumothorax incidence in CT-guided lung biopsy. Rofo.

[17] Pugliesi R.A., Schade I., Benchekroun A., BenAyed R., Mahnken A., Maalouf N., Apitzsch J. (2025). Reevaluating Routine Post-Biopsy Chest X-Rays After CT-Guided Lung Biopsy: Incidence of Pneumothorax and Value of Symptom-Based Monitoring. J. Clin. Med..

[18] Pugliesi R.A., Mahnken A.H., Maalouf N., Apitzsch J. (2025). Predicting Pneumothorax and Hemorrhage After CT-Guided Lung Biopsy: Role of Lesion Size, Depth and Their Interaction. J. Clin. Med..

[19] Sabatino V., Russo U., D’Amuri F., Bevilacqua A., Pagnini F., Milanese G., Gentili F., Nizzoli R., Tiseo M., Pedrazzi G. (2021). Pneumothorax and pulmonary hemorrhage after CT-guided lung biopsy. Radiol. Med..

[20] Huo Y.R., Chan M.V., Habib A.R., Lui I., Ridley L. (2019). Post-biopsy maneuvers to reduce pneumothorax incidence in CT-guided lung biopsy: A meta-analysis. Cardiovasc. Intervent. Radiol..

[21] Ho A.T.N., Gorthi R., Lee R., Chawla M., Patolia S. (2023). Solitary lung nodule: CT-guided biopsy vs transbronchial biopsy with EBUS. Lung.

[22] Gadaleta C.D., Iezzi R., Tanzilli A., Puppini G., Carriero P.L., Amato A. (2022). Pilot clinical study on the prevention of complications after lung biopsy by the MIPP kit PNX device. Transl. Cancer Res..

[23] Wagner J.M., Hinshaw J.L., Lubner M.G., Robbins J.B., Kim D.H., Pickhardt P.J., Lee F.T. (2011). CT-guided lung biopsies: Pleural blood patching reduces the rate of chest tube placement for postbiopsy pneumothorax. AJR Am. J. Roentgenol..

[24] Lang E.K., Ghavami R., Schreiner V.C., Archibald S., Ramirez J. (2000). Autologous blood clot seal to prevent pneumothorax at CT-guided lung biopsy. Radiology.

[25] Yamagami T., Nakamura T., Iida S., Kato T., Nishimura T. (2002). Management of pneumothorax after percutaneous CT-guided lung biopsy. Chest.

[26] Karmy-Jones R., Wood D.E. (2007). Traumatic injury to the trachea and bronchus. Chest Surg. Clin. N. Am..

[27] Kuriyama T., Masago K., Okada Y., Katakami N. (2018). Computed tomography-guided lung biopsy: Association between biopsy needle angle and pneumothorax development. Mol. Clin. Oncol..

[28] Chiu J.H., Chang Y.Y., Weng C.Y., Lee Y.C., Yeh Y.C., Chen C.K. (2022). Risk factors for pneumothorax and pulmonary hemorrhage following computed tomography-guided transthoracic core-needle biopsy of subpleural lung lesions. J. Chin. Med. Assoc..

[29] Saggiante L., Biondetti P., Lanza C., Carriero S., Ascenti V., Piacentino F., Shehab A., Ierardi A.M., Venturini M., Carrafiello G. (2024). Computed-Tomography-Guided Lung Biopsy: A Practice-Oriented Document on Techniques and Principles and a Review of the Literature. Diagnostics.

[30] Zhao Y., Xiong K., Lv Y.N. (2023). Low-dose CT-driven biopsy for pulmonary nodules: Systematic review. Videosurg. Other Miniinvasive Technol..

[31] Heerink W.J., de Bock G.H., de Jonge G.J., Groen H.J., Vliegenthart R., Oudkerk M. (2017). Complication rates of CT-guided lung biopsy: Meta-analysis. Eur. Radiol..

[32] Andrade J.R., Rocha R.D., Falsarella P.M., Rahal Junior A., Santos R.S.D., Franceschini J.P., Fernando H.C., Garcia R.G. (2018). CT-guided percutaneous core needle biopsy of pulmonary nodules smaller than 2 cm: Technical aspects and factors influencing accuracy. J. Bras. Pneumol..

[33] Han Y., Kim H.J., Kong K.A., Kim S.J., Lee S.H., Ryu Y.J., Lee J.H., Kim Y., Shim S.S., Chang J.H. (2018). Diagnosis of small pulmonary lesions by transbronchial lung biopsy with radial endobronchial ultrasound and virtual bronchoscopic navigation versus CT-guided transthoracic needle biopsy: A systematic review and meta-analysis. PLoS ONE.

[34] Lu Y., Wang Y., Ding Y., Chen D., He W., Zhong W., Yang J., Yan S., Ren G., Zhao F. (2025). Integrating peritumor and tumor CT radiomics features in predicting local control after SBRT in patients with pulmonary oligometastases. Radiat. Oncol..

[35] Badellino S., Cuccia F., Galaverni M., Miele M., Sepulcri M., Zerella M.A., Spoto R., Alì E., Olmetto E., Boldrini L. (2025). Lung Stereotactic Body Radiotherapy (SBRT): Challenging Scenarios and New Frontiers. J. Clin. Med..

[36] Yeow K.M., Tsay P.K., Cheung Y.C., Lui K.H., Pan K.T. (2004). Risk factors of pneumothorax and bleeding in 660 CT-guided biopsies. Chest.

[37] Grange R., Di Bisceglie M., Habert P., Resseguier N., Sarkissian R., Ferre M., Dassa M., Grange S., Izaaryene J., Piana G. (2023). Evaluation of preventive tract embolization with standardized gelatin sponge slurry on chest tube placement rate after CT-guided lung biopsy: A propensity score analysis. Insights Imaging.

[38] Young M., Sankari A. (2025). Percutaneous Lung Lesion Biopsy. StatPearls.

